# Emotion Analysis of Ideological and Political Education Using a GRU Deep Neural Network

**DOI:** 10.3389/fpsyg.2022.908154

**Published:** 2022-07-26

**Authors:** Shoucheng Shen, Jinling Fan

**Affiliations:** ^1^School of Marxism, Dalian Maritime University, Dalian, China; ^2^Office of Academic Affairs, Hebei University of Science and Technology, Shijiazhuang, China

**Keywords:** GRU, deep learning, political and ideological education, emotion analysis, feature fusion

## Abstract

Theoretical research into the emotional attributes of ideological and political education can improve our ability to understand human emotion and solve socio-emotional problems. To that end, this study undertook an analysis of emotion in ideological and political education by integrating a gate recurrent unit (GRU) with an attention mechanism. Based on the good results achieved by BERT in the downstream network, we use the long focusing attention mechanism assisted by two-way GRU to extract relevant information and global information of ideological and political education and emotion analysis, respectively. The two kinds of information complement each other, and the accuracy of emotion information can be further improved by combining neural network model. Finally, the validity and domain adaptability of the model were verified using several publicly available, fine-grained emotion datasets.

## Introduction

The emotional attributes of ideological and political education go hand-in-hand with those of ideology, society, practice, and culture. The unique features and causes of emotional attributes can be determined through the process of linking and distinguishing between them. Ideological and political education is based on the reality of people’s existences, including their emotions, which gives this form of education its distinctive emotional character. As a practical activity, ideological and political education deals with thinking human subjects, whose ideological and moral character it aims to improve. Emotion is not only fundamental to such aspects of character but also motivates the dialectical development of additional elements such as knowledge, intention, faith, and action. Ideological and political education aims to promote people’s all-round development, including their emotional development.

Recently, deep neural networks have increasingly been used in emotion analysis. GRU network is a simplified deformation model of long short-term memory (LSTM) first proposed by Ortigosa in 2014 (Ortigosa et al., 2012). The model simplified the three gate structures of the LSTM unit into two. The GRU network can retain long-distance sequence information, which means that these characteristic signals will not be weakened over time, nor will they be deleted due to irrelevant predictions. Peng proposed a gated convolutional network model based on convolutional neural networks (CNNs) and a gated mechanism (Peng et al., 2017). An attention mechanism in deep learning was first used in machine translation by Quadrana (Quadrana et al., 2018), and variations in this model were subsequently applied to emotion classification tasks. Gao proposed two attention mechanisms to improve model performance by considering the relationship between dimensional words and contextual semantic information (Gao et al., 2017).

With the continuous development and improvement of deep learning models, some scholars have combined multiple network structures to carry out emotion analysis research tasks. These have used the TF-IDF method to calculate feature weights and the support vector method to analyze the emotion, an approach that has delivered promising experimental results (Su and Li, 2013). The NBSVM hybrid model integrates the unique advantages of a support vector machine and naive Bayes algorithms, considerably improving the accuracy of emotion analysis compared with a single model (Kyunghyun et al., 2015). CNN and LSTM network structures have been used to classify emotions in comments on TikTok, also achieving good experimental results (Lopamudra et al., 2016). A bottom-up and end-to-end emotion analysis model combining CNN and RNN has also been proposed. Overall, this work demonstrates that approaches to emotion analysis based on deep learning offer higher levels of performance and accuracy (Liu et al., 2020) and point to their use in ideological and political education, ultimately supporting the emotional development of those who benefit from such learning opportunities.

This study investigated the application of end-to-end aspect-based emotion analysis (E2E-ABSA) to recommendation systems in ideological and political education. The study aimed to identify the most efficient and accurate model of emotion analysis, uncover further educational applications for emotion analysis, expand current approaches to text-based emotion analysis, and provide a durable theory to support its implementation.

## Improved Recurrent Neural Network Variant Theory and Technology

When training recurrent neural networks (RNNs), the gradient descent method may be used to counter gradient disappearance or explosion. Given this problem, many scholars have proposed variants in RNN neural networks, such as two-way LSTM and GRU networks. One method classified malware based on an RNN using API calling functions at an average of 71% accuracy. Comparisons of the effectiveness of GRU, backward GRU (BGRU), LSTM and backward LSTM networks, and regular RNN models for malware detection and classification showed that the backward LSTM model achieved the highest accuracy rate (97.85%). When malware samples were detected and classified using a combined model of a CNN and LSTM, the classification accuracy of the model reached 98.8%.

### Long Short-Term Memory

At present, the LSTM neural network architecture most widely used in practical applications is the model proposed by Hochreiter et al., which not only solves the problem of gradient disappearance in RNN but also performs much better than RNN in long sequence data-dependent tasks (Harper and Konstan, 2015) and avoids the problem of gradient disappearance during backpropagation. The network can accurately model any long-span-dependent data based on time series. While the basic principles behind LSTM and RNN are highly similar, LSTM networks can carry out more detailed internal processing and thus manage dependent information more effectively. Since the unique characteristics of LSTM neural networks are superior to those based on RNN, it is more widely used in time series-based learning-related tasks. LSTM aims to solve the long-term dependency problem mentioned above. Traditional RNN node outputs are determined only by weights, biases, and activation functions: RNN is a chain structure and each time slice uses the same parameters.

However, the LSTM model introduced a memory unit and gated memory unit, solving complex artificial long-delayed tasks, which had never been solved by previous recursive network algorithms.

In this study, the term *LSTM element* describes the hidden element in the LSTM recurrent neural network (LSTM). The LSTM architecture introduced in this study was originally proposed by Graves (Song et al., 2017).

**FIGURE 1:**
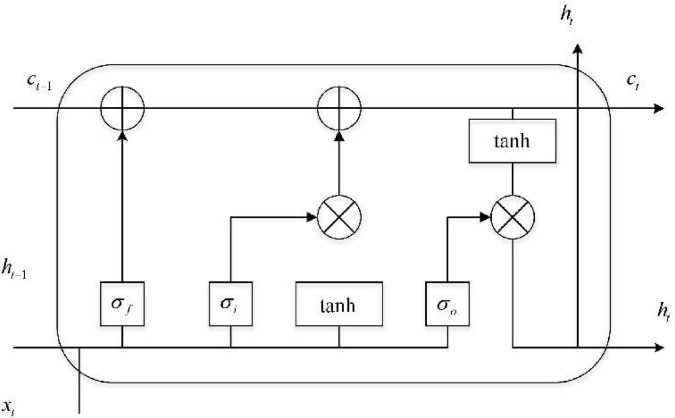
LSTM neural network structure.

The LSTM unit has three types of gating, as shown in Figure 1. The LSTM unit consists of input gate *i*, forget gate *f*, and output gate *o*. Gated units act as a full connection layer that enables the LSTM to store and update information. In more vivid and specific terms, the implementation of gating is the weight and sequence in the dot product when bias is added and is achieved through the sigmoid function. When internal information is sequenced, gating means that additional information is not provided. The mathematical expression of gating is shown below:


(1) g⁢(x)=σ⁢(W⁢x+b),

where σ(*x*) = 1/(1 + *e*^−*x*^) denotes the sigmoid function, the most frequently used non-linear activation function in machine learning, which is used to normalize data and map it to the interval [0,1]. The function of gate control is to describe how much information is passed. When *g*(*x*) = 0, all information is blocked and cannot be transmitted; when *g*(*x*) = 1, all information can be sent to the next unit. *W* and *b* represent the weight matrix and bias vector of the network, respectively.

The forward calculation process of the bidirectional long short-term memory network is expressed in Equations 2–6 below. At time T, the input of the hidden layer of LSTM is *x*_*t*_, the output vector of the hidden layer is *h*_*t*_, and its memory unit is *c*_*t*_. The function of the input gate is to control the amount of input data *x*_*t*_ and transfer them into memory units, i.e., amounts that can be saved into *c*_*t*_. Its mathematical expression is shown in the formula below:


(2) $$i_{t}=\sigma \,\left(W_{x\,i}\,X_{t}+W_{h\,i}\,h_{t-1}+b_{i}\right)$$

The forget gate is an important part of the LSTM neural network because it controls the degree to which information is forgotten; its mathematical expression is shown in Equation 3, below. It also avoids problems such as gradient disappearance and explosion caused by backpropagation. Forget gates determine which information is discarded based on the characteristics of historical information, that is, the influence of memory unit *c*_*t–1*_ at the previous moment on memory unit *c*_*t*_ at the present moment, and are expressed mathematically by the formula below (4):


(3) $$f_{t}=\sigma \,\left(W_{x\,f}\,x_{t}+W_{h\,f}\,h_{t-1}+b_{f}\right)$$

(4) $$c_{t}=f_{t}⊗c_{t-1}+i_{t}⊗\tanh\left(W_{x\,c}\,x_{t}+W_{h\,c}\,h_{t-1}+b_{c}\right)$$

The output value *h*_*t*_ of the output gate is related to the control memory unit *c*_*t*_. The output expression is shown in Equation 5 below and state at time *t* in Equation 6:


(5) $$o_{t}=\sigma \,\left(W_{x\,o}\,x_{t}+W_{h\,o}\,h_{t-1}+b_{o}\right)$$

(6) $$h_{t}=o_{t}⊗\tanh\left(c_{t}\right)$$

### Gated Loop Unit

**FIGURE 2:**
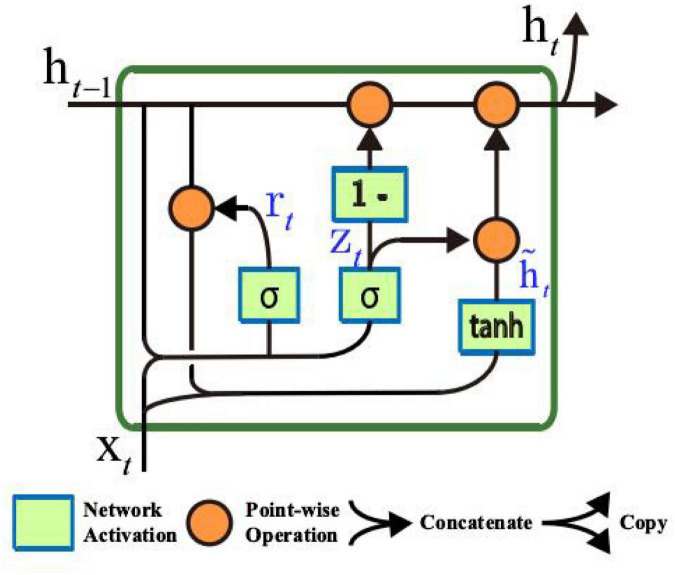
Gated recurrent unit (GRU).

A gated recurrent unit (GRU) is a network structure that uses multiple gated units to realize the flow, regulation, storage, memory, and other functions of information flow in the network (Li et al., 2019). In GRU, the activation of the gating unit depends only on the current input and the previous output. With fewer parameters and fast convergence, it can adaptively capture the dependence of different time scales. In some time-series data-processing tasks, GRU-based models are often superior to other network models (Mühlbacher et al., 2018). The specific internal structure of the gated recurrent unit GRU is shown in Figure 2.

The output of the gated loop unit GRU at time point *t* is $h_{t}=\left\{h_{t}^{1},\ldots ,h_{t}^{j},\ldots \right\}$, where *j* represents the *j* neuron, and $h_{t}^{j}$ is the linear interpolation between the output $h_{t-1}^{j}$ of the previous time point *t-1* and the candidate activation value ${\tilde{h}}_{t}^{j}$ of the current time point:


(7) $$h_{t}^{j}=\left(1-z_{t}^{j}\right)\,h_{t-1}^{j}+z_{t}^{j}\,{\tilde{h}}_{t}^{j}$$

where $z_{t}^{j}$ is the *j* unit of gate *z^t^*, and $z_{t}^{j}$ determines the update ratio of output unit $h_{t}^{j}$ of the GRU. The updated gating unit $z_{t}^{j}$ is calculated using the following formula:


(8) $$z_{t}^{j}=\sigma \,{\left(W_{z}\,x_{t}+U_{z}\,h_{t-1}\right)}^{j},$$

where *x*_*t*_ represents the input value at time *t*, $h_{t-1}={\left\{h_{t-1}^{1},\ldots ,h_{t-i}^{j},\ldots \right\}}^{j}$ and $h_{t-i}^{j}$ represent the *j* output unit at the last time point *t-1*. The method for calculating the candidate activation value ${\tilde{h}}_{t}^{j}$ is similar to the traditional regression unit:


(9) $${\tilde{h}}_{t}^{j}=\tanh{\left(Wx_{t}+U\left(r_{t}⊗h_{t-1}\right)\right)}^{j},$$

where $r_{t}=\left\{r_{t}^{1},\ldots \,r_{t}^{j},\ldots \right\}$ represents the reset gate, ⊗ represents the interelement (Hadamard) product, and tanh (⋅) represents the hyperbolic tangent function. The hyperbolic tangent function tanh (⋅) is defined as follows:


(10) $$\tanh\left(x\right)=\frac{e^{x}-e^{-x}}{e^{x}+e^{-x}}$$

The reset gate *r*_*t*_ is represented by the following formula:


(11) $$t_{t}^{j}=\sigma \,{\left(W_{r}\,x_{r}+U_{r}\,h_{t-1}\right)}^{j}$$

When the reset gate is closed (when $r_{t}^{j}$ has a value of 0), the reset gate effectively causes the GRU unit to behave as if it were reading the first symbol of the input sequence, causing the GRU to forget the previously calculated state. Using the update and reset gates, the gated loop unit GRU can realize the memory and update functions and adaptively capture the dependencies of different time scales.

### Recursive Neural Network Based on the Gated Loop Unit

**FIGURE 3:**
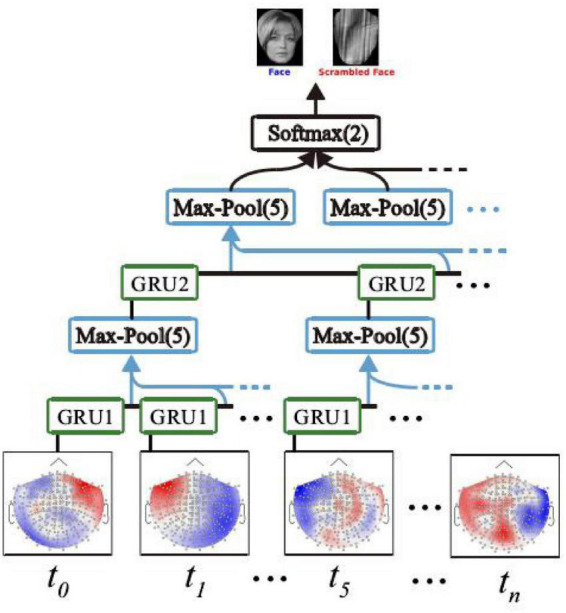
Recursive neural network based on gated loop unit GRU. Human image reproduced with permission from the TikTok dataset.

A recursive neural network based on the gated loop unit GRU can be built and applied to a MEG signal decoding task (Huo et al., 2016). The GRU-based recurrent neural network (GRNN) can adaptively capture the dependence of the MEG signal in different time scales, and its deep network structure enables complex MEG signal characteristics to be extracted (Han et al., 2018), thus decoding the signals effectively. Figure 3 shows a recursive neural network based on gated cyclic units, which consists of two GRU unit groups, two lower sampling layers, and an output layer.

In Figure 3, the two-layer GRU unit group includes GRU1 and GRU2, which are distributed on two different network layers. At each time point of the same network layer *t*_0_, *t*_*i*_., the GRU unit on *t*_*n*_ is shared. The sampling method of the lower sampling layer is max-pool (Hansen and Hasan, 2015). Max-pool (5) means that the maximum activation value at five time points is taken as the sampling value (Khan and Amjad, 2016). In addition, the output layer of the GRNN model is a fully connected layer with two output units, and the activation function of the output unit is the softmax function. The softmax function output can be used to generate prediction labels to determine whether the stimulus image corresponding to the current MEG signal is face or non-face.

The network can be trained and optimized by minimizing the cross-drops between the predicted and actual tags of the GRNN model, with the corresponding loss function as follows:


(12) $$L=-\sum_{c=1}^{2}y_{c}\,\log\left(p_{c}\right)+\left(1-y_{c}\right)\,\log\left(1-p_{c}\right),$$

where *c* = 1 represents the MEG signal induced by a face image, and *c* = 2, the MEG signal stimulated by a non-face image. *p*_1_ represents the probability of the GRNN model detecting a face-prompted MEG signal, and *p*_2_, the probability that a MEG signal induced by non-face images is detected. [*y*_1_,*y*_2_]*^T^* represents the actual label of the input signal, if the input signal is induced by face image stimulation, then [y_1_, y_2_]T = [1, 0]T, otherwise [y1,y2]=T[1,0]T.

## Emotion Analysis of Ideological and Political Education Integrating Gru and the Attention Mechanism

### Overall Framework of Emotion Analysis Model Based on Deep Learning

**FIGURE 4:**
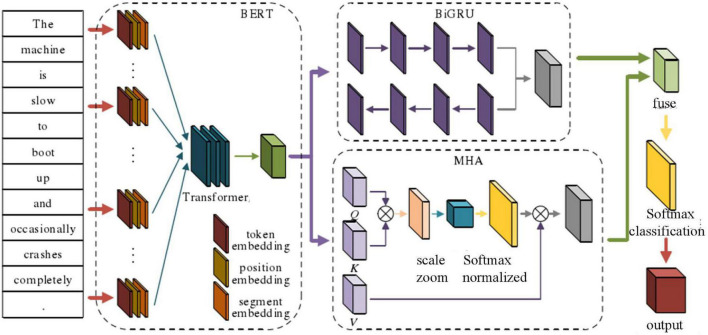
Overall architecture of E2E-ABSA model integrating GRU and MHA.

The emotion analysis model of ideological and political education based on deep learning includes BERT text processing, semantic feature extraction, and feature fusion calculation. The main model architecture is shown in Figure 4

A parallel approach was taken to the process of deep feature extraction. On the one hand, a bidirectional GRU network was adopted to solve the context-dependent problem and extract the associated semantics of similar texts. On the other hand, multi-head attention (MHA) based on the self-attention mechanism was taken as the main network (Herath et al., 2017). The aim was to capture the relationships and importance of each word in the text to achieve the overall capture of associated attributes and emotions; the feature fusion calculation link was inspired by the long attention mechanism concat operation and aimed to capture the characteristics of parallel deep features through integration in the form of weight, combining their advantages on the parallel network to comprehensively determine their characteristics. Finally, the softmax classification function was adopted to improve performance on the task of classifying and labeling text emotions.

### Text Feature Extraction Combining Gated Recurrent Unit and the Attention Mechanism

Following BERT processing to obtain the corresponding text feature vector representation *x* = (*x*_1_, *x*_2_, *x*_*n*_), the next step was to develop the deep network architecture for semantic parsing and understanding. The processes of text feature extraction, text relevance capture, and semantic representation have always been the primary tasks of deep learning neural networks; the richness of feature extraction determines the accuracy of subsequent classification tasks. Among numerous feature representation networks, GRU is renowned for its ability to capture context-related information and efficient training methods, enabling it to solve the problem of text-long dependence (Zhao et al., 2017). The multi-attentional mechanism has attracted much interest since being proposed. The multi-attentional parallel training method not only allows models to learn relevant information in different representation subspaces but also improves their learning performance and greatly reduces learning time, thereby improving the model’s overall efficiency. This study integrated GRU and the attention mechanism for the parallel extraction of text features.

#### Semantic Association Feature Representation in the Bidirectional Gated Recurrent Unit Network

The feature representation network needs to fully extract the expression of emotion words near feature attributes and identify their interactive relationship. The gating unit of the GRU network can capture long-distance relationships between elements of the text and can also represent the interaction between characters.

After the text vector *x* = (*x*_1_, *x*_2_, *x*_*n*_) passes through a GRU unit, the corresponding hidden layer output is $h_{t}=\left(1-z_{t}\right)*h_{t-1}+z_{t}*{\tilde{h}}_{t}$. Unidirectional GRU networks can capture the semantic information of the first half of the word, but not the second half. To deal with this constraint, GRU semantic capture networks imitating the bidirectional cascade of LSTM networks including two opposite directions (forward and backward) are widely adopted. After the forward and backward GRU units that traverse the sentence from the beginning and end of the text, respectively, the forward neuron hidden output ${\dot{h}}_{t}=\left({\dot{h}}_{1},{\dot{h}}_{2},\ldots \,{\dot{h}}_{n}\right)$ and the backward neuron hidden output $h_{t}=\left(1-z_{t}\right)*h_{t-1}+z_{t}*{\tilde{h}}_{t}$ can be obtained, and the two can be cascaded to obtain the coding output *y*_*t*_ of the bidirectional GRU network:


(13) $$y_{t}=\left[{\vec{h}}_{t},{\overset{\leftarrow }{h}}_{t}\right]=\sigma \left(W_{y\,\vec{h}}{\vec{h}}_{t}+W_{y\overset{\leftarrow }{h}}{\overset{\leftarrow }{h}}_{t}\right)$$

In the process of forward and backward propagation, all W parameters need to be learned. In this study, the backpropagation through time (BPTT) algorithm was adopted to continuously adjust and optimize the weight vector of the GRU network. Assuming the loss of a single sample at a certain time is *P*_*t*_ = (*y*_*d*_−*y*_*t*_)^2^/2, where *y*_*d*_ represents the true value and *y*_*t*_ represents the predicted value, the loss of the sample at all times is $E=\sum_{t-1}^{T}P_{t}$, and *T* represents the time sequence length. The partial derivative of the loss function to each weight parameter is obtained, and the loss convergence is achieved by successive generations that update the parameters:


$$\frac{\partial E}{\partial W_{y\,h}}=\delta_{y,t}\,h_{t}\,\left(4.2\right)\,\frac{\partial E}{\partial W_{z\,x}}=\delta_{z,t}\,x_{t}\,\left(4.3\right)$$

(14) $$\frac{\partial E}{\partial W_{z\,h}}=\delta_{z,t}\,h_{t-1}\,\left(4.4\right)\,\frac{\partial E}{\partial W_{h\,x}}=\delta_{t}\,x_{t}$$

(15) $$\frac{\partial E}{\partial W_{h\,h}}=\delta_{t}\,\left(r_{t}\cdot h_{t-1}\right)\,\left(4.6\right)\,\frac{\partial E}{\partial W_{r\,x}}=\delta_{r,t}\,x_{t}\,\left(4.7\right)\,\frac{\partial E}{\partial W_{r\,h}}=\delta_{r,t}\,h_{t-1}$$

The eight parameters are as follows:


(16) $$\left\{ \begin{array}{c} \delta_{y,t}=\left(y_{d}-y_{t}\right)\cdot \sigma^{\prime }\\ \delta_{h,t}=\delta_{y,t}\,W_{y\,h}+\delta_{z,t+1}\,W_{z\,h}+\\ \delta_{t+1}\,W_{h\,h}\cdot r_{t+1}+\delta_{h,t+1}\,W_{r\,h}+\delta_{h,t+1}\cdot \left(1-Z_{t+1}\right)\\ \delta_{z,t}=\delta_{t,h}\cdot \left({\tilde{h}}_{t}-h_{t-1}\right)\cdot \sigma^{\prime }\\ \delta_{t}=\delta_{h,t}\cdot z_{t}\cdot \phi^{\prime }\\ \delta_{r,t}=h_{t-1}\cdot \left[\delta_{h}\,W_{h\,h}\right]\cdot \sigma^{\prime } \end{array}\right.$$

The sequence vector *x* = (*x*_1_, *x*_2_, *x*_*n*_) is sent into the BiGRU network to extract corresponding features through BPTT algorithm training, and the corresponding state matrix is obtained. The specific calculation method is as follows:


(17) $$h_{t}=B\,i\,G\,R\,U\,\left(h_{t-1},h_{t+1},x_{t}\right)$$

and


(18) $$H=\left(h_{0},h_{1},\ldots ,h_{n}\right)\in R^{d*n},$$

where *d* represents the dimension of the output state matrix of the hidden layer, *d*∈*R*.

#### Multi-Attentional Network Based on the Self-Attentional Mechanism

Because different words in the same text vary in importance, weights must be allocated based on specific attributes. To accurately predict tags in the E2E-ABSA task, the model must be able to capture key information. Initially, the attention mechanism allocates more resources to model the key local information, and a more incisive and vivid long attention mechanism is developed. The parallel learning mechanism enables different dimensions of multi-angle text calculations to be made.

**FIGURE 5:**
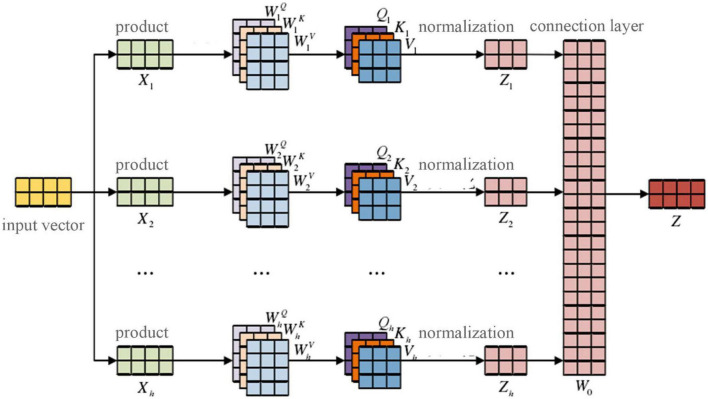
Semantic feature extraction based on the multi-attentional mechanism.

In this study, a self-attention-based multi-attentional mechanism was added after BERT text representation to calculate correlations and assign importance to the words used in each sentence of the text. The process of extracting volume semantic features is shown in Figure 5, below.

The input vector *x* = (*x*_1_, *x*_2_, *x*_*n*_) is mapped to *h* subspace, that is, the multi-headed attention mechanism has *h* heads, and the corresponding dimension representation *X*_1_,*X*_2_,…,*X*_*n*_ is obtained by


(19) $$X_{g}=x\to X^{a\times b},$$

where *a* × *b* is the dimension of the corresponding subspace *g*, *g* ∈ [1,*h*].

The corresponding initialization *Q_*g*_, K_*g*_, V_*g*_* and the vector are calculated as follows:


(20) $$Q_{g}=W_{g}^{Q}*X_{g},K_{g}=W_{g}^{K}*X_{g},V_{g}=W_{g}^{V}*X_{g},$$

where $W_{g}^{Q}$, $W_{g}^{K}$, and $W_{g}^{V}$ are the appropriate parameters learned by the model in the training process. After the corresponding attention calculation, the attention output *Z*_*g*_ of each subspace is follows:


(21) $$Z_{g}=s\,o\,f\,t\,\max\left(\frac{Q_{g}\,K_{g}^{T}}{\sqrt{d_{k}}}\right)\,V_{g}.$$

The attention vectors of each subspace are concat-spliced to form a combined multi-head attention matrix, and the final semantic parsing output is obtained by calculating the weight matrix for the fully connected layer (Bai et al., 2016):


(22) $$Z_{a\,l\,l}=c\,o\,n\,c\,a\,t\,\left(Z_{1},Z_{2},\cdots ,Z_{h}\right)$$

and


(23) $$Z=Z_{a\,l\,l}*W_{0},$$

where W_0_ is the weight vector of the full connection layer.

#### Feature Fusion and Calculation

After obtaining the semantic representation of the BiGRU and multi-attention feature extraction networks, the matrix representation of the two is merged and calculated in the next step to achieve multi-angle semantic capture of the text combining their representational advantages (Adeniyi et al., 2016). To fully retain the original acquisition characteristics of BiGRU and MHA, this study adopted a simple matrix weight splicing method to merge them. The specific calculation is as follows:


(24) O=(1-μ)*Z+μ*H,

where *O* is the final feature output vector representation of the text statement, μ is the feature reserved weight of the BiGRU network, and its initial value is set artificially and continuously adjusted automatically in the model learning process.

The output *O* = (*o*_1_, *o*_2_, …, *o*_*n*_), with the softmax function used for classification calculations is


(25) $$P\,\left(y\,t|o_{t}\right)=s\,o\,f\,t\,\max\left(W_{o}\,o_{t}+b_{o}\right),$$

where *P*(*yt*|*o*_*t*_) represents the probability of *y*_*t*_ output when the feature vector is input *o*_*t*_, and *o*_*t*_ is the *t* vector of *O*.

### Test Process and Analysis of Results

The TikTok and network service evaluation datasets were added on the basis of the Laptop14 and Rest14 sets. A horizontal comparison of the classical depth feature extraction network was carried out on the TikTok dataset, and the service dataset was compared vertically, layer-by-layer (Jiang et al., 2017). The results of Laptop14 and Rest14 were compared to some advanced benchmark models. All datasets were annotated with supplementary sequences and applied to the emotion analysis model after label transformation, which further verified the stability and superiority of the E2E-ABSA model combined with GRU and the attention mechanism in multi-domain text learning.

#### Test Setting and Parameter Selection

The BiGRU network was set as a single-layer bidirectional cascade. The platform configuration and parameter settings of the BERT model processing link in the depth model integrating BiGRU and MHA are shown in Table 1.

**TABLE 1:** Parameter settings related to the model.

Related network dimensions and parameter meanings	The specific Settings
Number of heads for multiple attention mechanisms	*n* = 12 dropout = 0.1
Initial value of BiGRU feature weight parameter μ	μ = 0.5
Initial learning rate of model	Learning_rate = 2e^–5^
Multiple attention network packet loss rate	Dropout = 0.1
Unidirectional GRU network dimension	Hidden_size = 384
Multidimensional attention network dimensions	dim_a_ = 768

#### Construction of Multi-Attention Network Based on the Self-Attention Mechanism

The TikTok dataset contains ten cross-datasets, each of which is divided into training and test sets. Since the original text of the ten sets is basically identical, we randomly selected one to serve as the experimental dataset for the model. In total, 10% of the total data in the training and test sets were randomly extracted from the training set to form the verification set. The specific distributions of semantic aspects in these datasets are shown in Table 2.

**TABLE 2:** Specific distributions of semantic aspects in the TikTok datasets.

Dataset partitioning	Sentence	Aspect		
		Positive	Negative	Neutral
Training set	1880	547	219	1799
Validation set	235	81	24	230
Test set	235	75	31	236
Total	2350	703	274	2265

**FIGURE 6:**
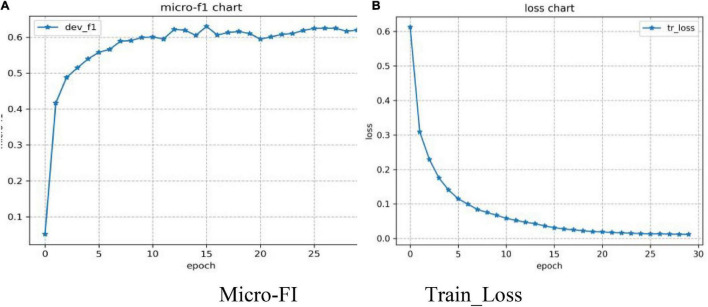
Changes in the BERT-BiGRU-MHA model on the TikTok dataset. **(A)** Micro-FI; **(B)** Train_Loss.

A total of four evaluation indexes (Micro-P, Micro-R, Micro-F1, and Macro-F1) were used to evaluate the model. A batch size of 32 was set for training on the TikTok dataset. Figure 6 shows that the model adding GRU and the attention mechanism feature extraction network based on BERT maintained fast convergence speeds and high accuracy across 30 epochs of the training process.

To verify the validity of the BERT-BiGRU-MHA model, feature extraction was performed in combination with the classic bidirectional long short-term memory network (BiLSTM) and CRF model using the BERT-based model. A horizontal comparison was made using the depth model that integrates BiGRU and MHA. The experimental results are shown in Table 3.

**TABLE 3:** Comparison between the experimental results of models based on the TikTok dataset.

Model	Micro-P	Micro-R	Micro-F1	Macro-Fl
BERT-Linear	0.5969	0.5774	0.5869	0.4491
BERT-BiLSTM	0.6024	0.5952	0.5988	0.3525
BERT-CRF	0.6152	0.6042	0.6096	0.4691
BERT-MHA	0.6399	0.5923	0.6151	0.4691
BERT-BiGRU	0.6228	0.6190	0.6208	0.4627
BERT-BiGRU-MHA	0.6449	0.6161	0.6301	0.4846

Table 3 shows that the emotion analysis models using BiLSTM and CRF scored more highly on the Micro-F1 index compared with BERT-Linear (the benchmark model lacking other feature extraction networks), indicating they were capable of representing the sequence of features. However, the Macro-F1 index of the BERT-BiLSTM model was 9.66% lower than the benchmark. It thus struggled to extract the features of less distributed categories, producing disappointing results in the search and classification accuracy of small sample category labels. At the same time, the Micro-F1 result for the BiGRU model was 2.20% higher than that of the BiLSTM. Thus, although BiGRU and BiLSTM would be expected to perform in similar ways, the performance of BiGRU on the actual E2E-ABSA task was much stronger.

It can be seen from the analysis of data distribution in Table 3 that the TikTok dataset suffers from a serious sparsity of data, whose distribution varies greatly between different emotional polarities. There was a large quantity of neutral emotional data, whereas obviously, positive and negative emotions were much less common. The BERT-BiGRU-MHA model data show that its results against the four indicators of Micro-P, Micro-R, Micro-F1, and Macro-F1 were 4.8, 3.87, 4.32, and 3.55% higher, respectively, than those of the BERT-Linear model. Moreover, its results for the Micro-F1 indicator were 3.13 and 2.05% higher than the traditional BERT-BiLSTM and BERT-CRF models, respectively. These results confirm that the feature extraction fusion model proposed in this study is superior to the traditional single feature extraction model and can fully adapt to uneven data distribution while simultaneously performing the dual representation of text attributes and emotions. As a result, the correlations between two words can be identified, enabling their interrelationships to be understood from a deeper perspective.

#### Experimental Results and Analysis of the Service Dataset

The service dataset is used less frequently than other sets for the E2E-ABSA task, mainly because its data are initially used for sentence-level and subject-related tasks. However, it was used to expand the scope of the application of the model presented in this study. The effects of the GRU network and multiple attention on feature extraction were verified longitudinally. The specific distributions of semantic aspects in the service datasets are shown in Table 4.

**TABLE 4:** Specific distributions of semantic aspects in the service datasets.

Dataset partitioning	Sentence	Aspect		
		Positive	Negative	Neutral
Training set	1343	936	620	100
Validation set	149	98	78	12
Test set	747	506	320	61
Total	2239	1540	1018	173

The depth feature extraction network was added layer-by-layer based on the BERT model to verify the depth model’s adaptability to datasets from different fields (refer to results in Table 5).

**TABLE 5:** Comparison between the experimental results of models based on the service dataset.

Model	Micro-P	Micro-R	Micro-F	Macro-Fl
BERT-Linear	0.6428	0.6268	0.6347	0.4778
BERT-BiLSTM	0.6471	0.6589	0.6359	0.5138
BERT-BiGRU	0.6570	0.6392	0.6479	0.4878
BERT-BiGRU-MHA	0.6585	0.6347	0.6463	0.4902

In terms of overall performance indicators, the BERT-MHA and BERT-BiGRU models performed similarly when locating and classifying most tags, but there was a 2.6% difference in performance on the Macro-F1 indicator, demonstrating that the capture of key information essential for attribute extraction and emotion classification is more important than capturing long-distance information in sparsely-distributed tag categories. Overall, however, the BERT-BiGRU-MHA model outperformed the benchmark, indicating that fusing the two improved the analysis of emotion in the service dataset.

Taking the most important index (Micro-F1) as the starting point, the BERT-MHA model with a multi-attentional mechanism and the BERT model combined with the BiGRU model in the downstream network improved on the BERT-Linear benchmark model by 1.24 and 1.32%, respectively. These results indicate that both the multi-attentional mechanism and GRU network can capture deep features and compute their semantic relevance very effectively. However, the Micro-F1 index for BERT-BİGRU-MHA shows that it did not outperform the separate feature extraction models BERT-MHA and BERT-BiGRU. This result is primarily because the service dataset contains an insufficient quantity of overall text and attributable emotions. As the model’s complexity increases, a greater quantity of data are required and only sufficient training can make the model fit completely. Thus, while the attention mechanism and feature extraction of datasets provided by GRU are sufficient, the increased complexity of the fusion model requires more modeling and data to comprehensively learn the characteristics of the domain. This explains why the index values for the basic and fusion models were similar. Future research may consider how to improve the adaptability of complex models with relatively small datasets.

#### Experimental Results and Analysis of the Laptop14 and Rest14 Datasets

The experimental results for the two datasets were compared with those of other advanced baseline models to conduct quantitative and qualitative analyses of the models. The experimental results of this process are shown in Table 6.

**TABLE 6:** Primary experimental results of each model on the Laptop14 and Rest14 datasets.

Model	Laptop14			Restl4		
	Micro-P	Micro-R	Micro-F1	Micro-P	Micro-R	Macro-Fl
MNN (2021) (Yahya et al., 2017)	0.5966	0.5733	0.5830	0.6678	0.6952	0.6793
DOER (2021) (Poria et al., 2016)	0.6012	0.5814	0.6035	0.6752	0.7019	0.7278
DREGCN-CNN-BERT (2022) (Boll, 2017)	0.6205	0.6071	0.6304	0.6933	0.7091	0.7260
BERT-Linear	0.6135	0.5883	0.6006	0.6997	0.7196	0.7095
BERT-BiGRU	0.6047	0.6104	0.6075	0.7140	0.7312	0.7225
BERT-MHA	0.6231	0.5868	0.6043	0.7397	0.7080	0.7235
BERT-BiGRU-MHA	0.6500	0.6151	0.6320	0.7543	0.7375	0.7458

(1) MNN. This model uses a multi-task neural network (MNN) to solve the end-to-end unified text emotion analysis task. A combined CNN-BiLSTM network equipped with the self-attention mechanism was used to capture the relationship between attributes and emotions. The interactive attention mechanism was integrated with the feature output of the combined network to analyze emotions without external language resources on a unified sequence annotation dataset.
(2) Dual cross-shared RNN (DOER). Similar to the RNN network stack utilization in reference, the DOER model is based on dual cross-shared RNN for emotion analysis. The residual network connected the GRU network, and a cross-sharing unit was set to carry out interactive calculations of attributes and emotions.
(3) DREGCN-CNN-BERT. Based on the DREGCN model in reference, the CNN network and BERT were added to extract additional local and global features.

Compared with the MNN model, the BERT-based model achieved Micro-F1 improvements of 1.76 and 3.02% on the Laptop14 and Rest14 datasets, respectively. Thus, compared to the multi-task network model, the unified task model structure is not centralized and the end-to-end performance is relatively poor. The DOER and BERT-Linear models performed at similar levels, further confirming the former’s powerful text-processing capability. However, when GRU and the attention mechanism were added to BERT, the effect was significantly improved, fully verifying the analysis they had performed. The MNN model based on the attention mechanism and the DOER model using GRU performed better than the BERT-MHA and BERT-BiGRU models in this study. This indicates that the combination of BERT, GRU, and the multi-attention mechanism may maximize their respective advantages in the feature network. The DREGCN-CNN-BERT and BERT-BiGRU-MHA models recorded similar values against the indicators on the Laptop14 dataset. On Rest14, the Micro-F1 values for the BERT-BiGRU-MHA model reached 74.58%, confirming its superiority over CNN for extracting local information. The context-related features of GRU and the multi-attention mechanism complemented the global features of this dataset more effectively.

**FIGURE 7:**
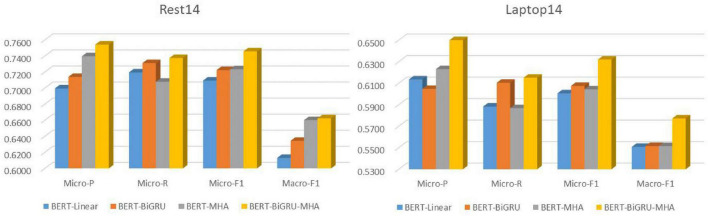
Comparison of corresponding performance indexes of each model.

For the Laptop14 and Rest14 datasets, the BERT-MHA and (particularly) BERT-BiGRU models improved on the Micro-F1 values recorded for the benchmark BERT-Linear model by 3.14 and 3.63%, respectively. Compared with the single feature extraction model, the fusion model of the Laptop14 and Rest14 sets reached 63.20 and 74.58% on Micro-F1, with average improvements of 2.61 and 2.28% further confirming the superiority of the fusion model. The Micro-*P* values were higher than those of Micro-R in both datasets for the BERT-BiGRU-MHA model, with the Micro-P 2.585% higher than the Micro-R on average, indicating that the model’s ability to make accurate predictions is higher than its tag-searching ability (refer to Figure 7).

There was a clear difference in the effect of MHA and BiGRU on the two datasets. The BERT-MHA model with attentional mechanism produced significant improvements in Micro-P, indicating that the mechanism can screen and capture key information. Meanwhile, the BERT-BiGRU model combined with GRU had a clear effect on Micro-R, indicating the effectiveness of GRU for extracting associated text semantics. The integrated network fully combines the advantages of both and performed better on all indicators, including Macro-F1.

Overall, GRU helped with the attention mechanism and while the fusion feature extraction of the network on small datasets requires a certain amount of fitting, the labels were found and the classification was accurate. This performance on the E2E-ABSA task demonstrated its potential use as a backbone network for extracting text semantics and contextual relevance.

## Conclusion

Text emotion analysis offers an intelligent and efficient means of locating hidden information in large-scale data and is an effective way for machines to “read” people. It has great potential in application fields such as recommendation systems. this study aimed to optimize a model for analyzing the emotional depth of ideological and political education. The analysis can be adapted to a variety of subtasks, including the exploration of dispersed emotion and the theory and practical application of cohesion in texts. It can also be used to analyze the emotional content of personalized recommendation systems and the convergence properties test. It is capable of deeper, end-to-end, and fine-grained exploration of the emotional content of texts. Using BERT text processing, a feature extraction depth model combining a bidirectional GRU network with a multi-attentional mechanism is proposed, constructing a complete E2E-ABSA model framework. Focusing on a deep learning model of text emotion analysis, this study has introduced the feature extraction principle of BiGRU and the method of its realization using the multi-attention mechanism and has expounded the calculations for fusing them. Second, a comparative test of the model was conducted on multiple datasets to analyze the role of each network and the effectiveness of the whole model.

## Data Availability Statement

Publicly available datasets were analyzed in this study. This data can be found here: https://github.com/IsakZhang/Generative-ABSA.

## Author Contributions

SS: manuscript writing and the experiments. JF: supervision. Both authors contributed to the article and approved the submitted version.

## Conflict of Interest

The authors declare that the research was conducted in the absence of any commercial or financial relationships that could be construed as a potential conflict of interest.

## Publisher’s Note

All claims expressed in this article are solely those of the authors and do not necessarily represent those of their affiliated organizations, or those of the publisher, the editors and the reviewers. Any product that may be evaluated in this article, or claim that may be made by its manufacturer, is not guaranteed or endorsed by the publisher.
